# Impact of respiratory motion on breast tangential radiotherapy using the field-in-field technique compared to irradiation using physical wedges

**DOI:** 10.2478/raon-2013-0032

**Published:** 2014-01-22

**Authors:** Hidekazu Tanaka, Shinya Hayashi, Kazuhiro Ohtakara, Hiroaki Hoshi

**Affiliations:** Department of Radiology, Gifu University Hospital, Yanagido 1-1, Gifu, Japan

**Keywords:** breast cancer, radiotherapy, field-in-field technique, respiratory motion

## Abstract

**Background:**

This study aimed to evaluate whether the field-in-field (FIF) technique was more vulnerable to the impact of respiratory motion than irradiation using physical wedges (PWs).

**Patients and methods:**

Ten patients with early stage breast cancer were enrolled. Computed tomography (CT) was performed during free breathing (FB). After the FB-CT data set acquisition, 2 additional CT scans were obtained during a held breath after light inhalation (IN) and light exhalation (EX). Based on the FB-CT images, 2 different treatment plans were created for the entire breast for each patient and copied to the IN-CT and EX-CT images. The amount of change in the volume of the target receiving 107%, 95%, and 90% of the prescription dose (V107%, V95%, and V90%, respectively), on the IN-plan and EX-plan compared with the FB-plan were evaluated.

**Results:**

The V107%, V95%, and V90% were significantly larger for the IN-plan than for the FB-plan in both the FIF technique and PW technique. While the amount of change in the V107% was significantly smaller in the FIF than in the PW plan, the amount of change in the V95% and V90% was significantly larger in the FIF plan. Thus, the increase in the V107% was smaller while the increases in the V95% and V90% were larger in the FIF than in the PW plan.

**Conclusions:**

During respiratory motion, the dose parameters stay within acceptable range irrespective of irradiation technique used although the amount of change in dose parameters was smaller with FIF technique.

## Introduction

Most patients with early stage breast cancer are administered breast-conserving treatment consisting of wide excision and postoperative whole breast radiotherapy. This form of postoperative radiotherapy reduces the risk of local recurrence and results in a long-term survival similar to that obtained with mastectomy.^1^–^3^

In recent years, the field-in-field (FIF) technique has become a widely performed method of administering tangential whole breast radiotherapy, in addition to the use of physical wedge (PWs). Several studies have reported that the use of the FIF technique allows for the better control of dose homogeneity.^4^–^9^ However, as the FIF technique requires the precise setting of the position of the multi-leaf collimators (MLCs) in order to reduce hot spots, there is concern that its use could significantly change the dose distribution to the target volume due to respiratory motion. The purpose of this study was to evaluate whether the FIF technique is more vulnerable to the impact of respiratory motion than irradiation using PWs.

## Patients and methods

This planning study included 10 patients with early stage breast cancer, 6 with right-sided, and 4 with left-sided breast cancer. All patients had undergone breast-conserving surgery and implantation of 4 surgical clips on the tumor bed, 2 of which had been placed in the nipple side of the tumor bed and 2 on each medial and lateral side of the tumor bed.

### CT acquisition

Computed tomography (CT) images were obtained using a scanner with 16 detector arrays (LightSpeed Xtra; GE Healthcare, Waukesha, WI, USA) while patients were in the supine position on a breast board with both arms above their heads. After radiopaque markers had been placed at the midline, the mid-axillary line, a site 1 cm below the infra-mammary fold, and at the level of the head of the clavicle, scanning was performed in 2.5-mm slices from the clavicle to the mid-abdomen during free breathing (FB). After the acquisition of the FBCT data set, 2 additional CT scans were obtained during a held breath after a light inhalation (IN) and a light exhalation (EX). All CT images were transferred to Eclipse External Beam Planning 6.5 software (Varian Medical Systems Palo Alto, CA, USA). We fused the IN-CT and EX-CT images with the FB-CT images according to the spine. Images fusion was easy and very precise because the CT scans were obtained continuously without any movement of the body.

### Simulation of radiotherapy planning

The remaining whole breast was contoured as the clinical target volume (CTV) with reference to the radiopaque markers. The planning target volume (PTV) was defined as the CTV with 5-mm margins except for the skin area. The evaluated planning target volume (PTVeval) was edited 5-mm of the build-up region from the skin surface of the breast. PTV defined on IN-CT and EX-CT was copied from FB-CT. The delineation was then moved and corrected on each CT slice, if necessary. The PTVeval on IN-CT or EX-CT was edited 5-mm of the buildup region from the skin surface of the PTV.

**FIGURE 1.**
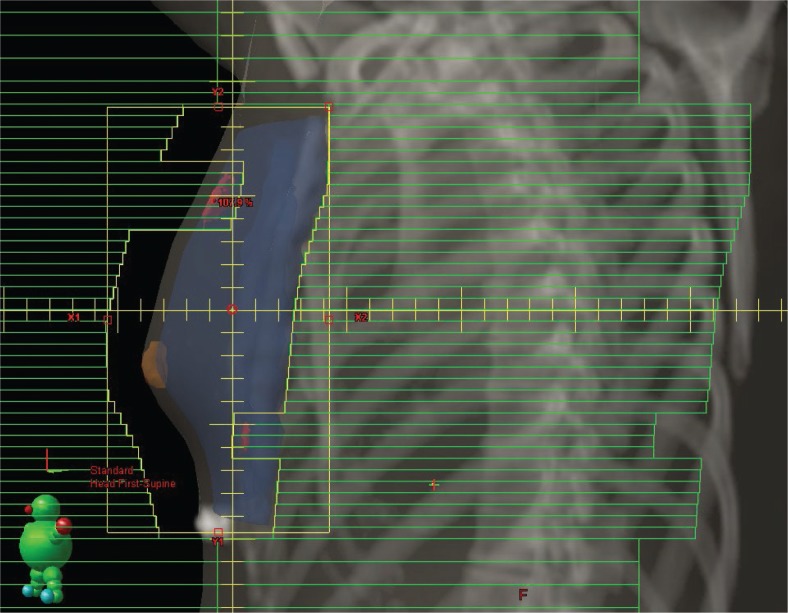
The additional subfield was designed to shield the hot region receiving ≥ 107% of the prescription dose. The evaluated planning target volume (PTVeval) is shown in dark blue, the nipple in brown, and the 107% isodose cloud in red.

The FB-CT images were used as references for specifying the beam arrangement for developing a conventional PW plan and an FIF plan. After performing an initial calculation with a tangential 6-MV photon beam, the gantry angles and dorsal borders of the tangential field were adjusted such that the central lung distance (CLD) was < 2cm. CLD is defined as the distance between the deep field edge and the interior chest wall at the central axis.^10^ Each patient’s plan was normalized to a reference point set as the midpoint of the nipple and the posterior border of the field. None of the reference points was located on the lung parenchyma or the border between the lung and chest wall. The prescribed dose was 50 Gy in 25 fractions. The dose calculation algorithm used was according to the pencil-beam convolution method, and the Batho power-law method was used to correct for tissue inhomogeneities. After beam weighting had been optimized for each case, the medial field was copied as the subfield. The MLCs of the subfield were manipulated to shield the areas of the breast receiving doses ≥ 107% of the prescription dose on beam’s eye view (Figure 1), with the beam weight of the subfield set at approximately one-tenth of the main field. If areas receiving a dose > 107% remained after recalculation of the dose distribution, the same process was repeated using a lateral field. All additional subfields were set not to shield the reference point.

After copying these fields to the IN-CT and EXCT images for each patient, dose calculation was performed by inputting the same number of monitor units as for the FB-plan. The PW plan was then created by adding the PWs to the initial open field plan on the FB-CT images. The angles of the PWs were ranged from 15° to 30°. This plan was also copied to the IN-CT and EX-CT images for each patient for dose calculation by inputting the same number of monitor units as for the FB-plan.

### Evaluation

A dose-volume histogram (DVH) was calculated for each patient and the volumes of the PTVeval receiving 107%, 95%, and 90% of the prescription dose (V107%, V95%, and V90%, respectively) were calculated. The homogeneity index (HI) was defined as HI = (D2 - D98) / Dprescription, where D2 is the dose given to 2% of the PTVeval, D98 is the dose given to 98% of the PTVeval, and Dprescription is the prescription dose. The maximum, mean, and minimum doses delivered to each surgical clip were also calculated. The amount of change in the IN-plan and EX-plan from the FB-plan were evaluated for both the FIF and PW plans. Dosimetric parameters were compared using the Wilcoxon signed-rank test. A *p* value less than 0.05 was considered to indicate a statistically significant difference. The length of movement of each surgical clip from EX-CT to IN-CT in 3 directions (horizontal, anteroposterior, and craniocaudal) and three-dimensional vector displacement were measured.

## Results

The median age of the patients was 54 years (range, 47 to 66 years). As shown in Table 1, which lists the displacement lengths of the clips in each direction, the average displacement length was the largest in the anteroposterior direction and the average three-dimensional vector displacement was 7.4mm.

No statistical differences were found regarding the amount of change for each surgical clip according to dose distribution between the IN-plan and FB-plan, or between the EX-plan and FB-plan.

**FIGURE 2.**
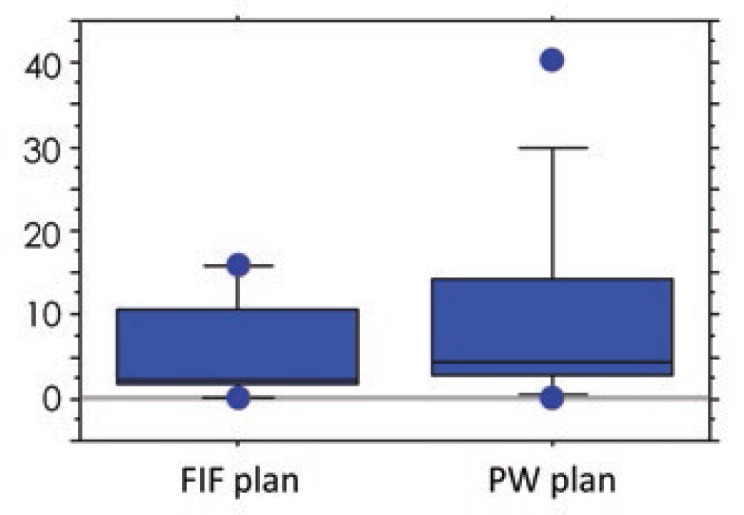
Comparison of amount of change of in the volume of the target receiving 107% (V107%) from the free breathing plan (FB-plan) to the light inhalation plan (IN-plan) for the field-in-field (FIF) and physical wedges (PW) plans.

**FIGURE 3.**
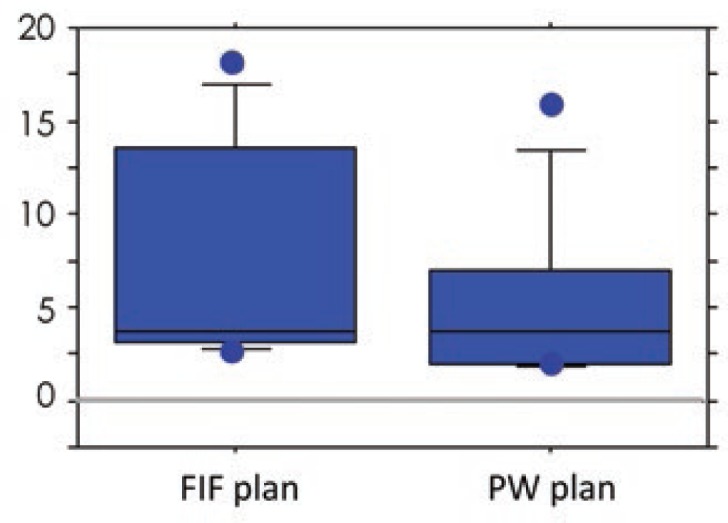
Comparison of amount of change in the volume of the target receiving 95% (V95%) from the free breathing plan (FB-plan) to the light inhalation plan (IN-plan) for the field-in-field (FIF) and physical wedges (PW) plans.

**FIGURE 4.**
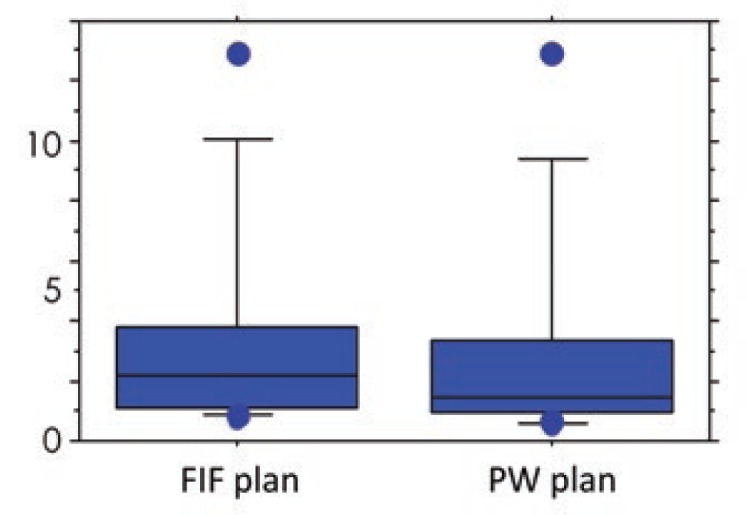
Comparison of amount of change in the volume of the target receiving 90% (V90%) from the free breathing plan (FB-plan) to the light inhalation plan (IN-plan) for the field-in-field (FIF) and physical wedges (PW) plans.

The V107%, V95%, and V90% of the IN-plan were significantly larger in both the FIF and PW plans than those of the FB-plan (Table 2). The mean amount of change in the V107% of the FIF and PW plans was 5.7% (range, 0 − 16.0%) and 9.8% (range, −0.1 − 40.3%), respectively. The amount of change in the V107% was significantly smaller in the FIF than in the PW plan (*p* = 0.0069; Figure 2). The amount of change in the V95% in the FIF and PW plans was 7.3% (range, 2.7−18.1%) and 5.4% (range, 1.8−15.8%), respectively. The amount of change in the V90% in the FIF and PW plans was 3.6% (range, 0.7−13.0%) and 3.1% (range, 0.5−12.9%), respectively. The amounts of change in the V95% and V90% were significantly larger in the FIF than in the PW plan (*p* = 0.0125 and 0.0093, respectively; Figure 3 and Figure 4). These findings indicate that the increase in the V107% was smaller and the increase in the V95% and V90% was larger in the FIF than in the PW plan. The V95% and V90% of the FB-plan were slightly small. The dorsal borders of the tangential field were adjusted so that the central lung distance was < 2 cm on the FB plan. In some cases, the dorsal part of the PTVeval was out of the irradiation field. The plan was approved with a confirmation that the remaining mammary tissue was covered by the 95% isodose line. In the IN plan, the thoracic wall was moved to the anterior and was included to a greater extent in the irradiation field. Although the PTVeval parameters were better, the irradiated lung volume increased. No significant differences between the FIF and PW plans were found regarding other parameters, including the HI. No significant differences in V107%, V95%, and V90% were noted between the FB-plan and EX-plan in both the FIF and PW plans. No significant differences were found regarding the amount of change in any parameter.

## Discussion

In a study of the effect of respiratory motion on breast tangential radiotherapy, Furuya *et al*. reported that movement along the anteroposterior direction significantly impacts dose distribution.^11^ In the current study, the average length of movement of the surgical clips was 7.4 mm and largest movement was in the anteroposterior direction.

The FIF technique has been reported to be a useful method of breast tangential radiotherapy. Compared to the use of open-field irradiation with or without a PW, the use of the FIF technique allows for a reduction in the size of the high-dose region and the HI.^4^–^9^ It has also been reported that it allows for the reduction of dosage to the contralateral breast.^9^ However, as the FIF technique requires the precise setting of the position of the MLCs in order to reduce hot spots, there is a concern that its use could significantly change the dose distribution to the target volume because of respiratory motion. Despite this concern, a few reports have evaluated the effect of respiratory motion on breast tangential radiotherapy using the FIF technique. Nakamura *et al*. simulated each FIF and PW plan based on FB-CT for 20 breast cancer patients, and then moved the plans posteriorly and recalculated the dose.^12^ They reported that the amount of change in the dose received by 98% of the PTV was smaller in the FIF than in the PW plan. However, as their simulation imitated respiratory motion, the deformation of thorax was not considered. To evaluate the effect of respiratory motion on an FIF plan, Bedi *et al*. created an FIF plan for 10 breast cancer patients on FB-CT images, copyied to the maximum inhalation and exhalation images obtained from four-dimensional CT.^13^ They found that, compared to the reference plan, D2, the V95% and V90% of the PTV had been increased during the inhalation phase and D2, the V95% and V90% had been decreased during the exhalation phase, but identified no significant difference in any parameters. The FIF plan was not compared with the PW. In a study that performed scanning for 10 breast cancer patients during 3 different phases (FB, IN, and EX) and then created FIF and PW plans, Frazier *et al*. reported that the V90%, V95%, and V100% for the ipsilateral breast were similar for each breathing position, but they did not statistically analyze their findings, or examine the difference in the amount of change with the use of the PW plan.^14^ To the best of our knowledge, the current study was the first to compare the amount of change due to respiratory motion when using an FIF plan and a PW plan for breast tangential radiotherapy by examining 3 different CT phases. The results revealed that the V107%, V95%, and V90% of the IN-plan were significantly larger than those of the FB-plan in both the FIF and PW plans, while the increase in V107% was smaller and the increase in V95% and V90% was larger in the FIF than in the PW plan. Thus, the increase in the size of the “hot region” was smaller and the decrease in the size of the “cold region” was larger in the FIF plan than in the PW plan. However, no significant differences were found between the plans regarding the amount of change in the HI, which we hypothesized, may have been due to the small number of cases examined.

In conclusion, the results of this study indicate that the amount of change in dose parameters due to respiratory motion was smaller with the FIF technique than with irradiation using physical wedges, within an acceptable range.

## Figures and Tables

**TABLE 1.** Displacement lengths of surgical clips from exhalation CT to inhalation CT

	**Displacement length (mm)**	**Standard deviation**	**Minimum (mm)**	**Maximum (mm)**
From lateral to medial	0.1	1.90	−3.3	4.2
From posterior to anterior	6.4	3.50	1.4	12.0
From caudal to cranial	2.7	2.40	−2.5	5.0
Three-dimensional vector	7.4	3.80	1.7	12.8

**TABLE 2.** Mean dose delivered to the evaluated planning target volume using the field-in-field and physical wedges plans during free breathing and light inhalation

		**FB**	**IN**	**p value**
FIF	V107%	0	5.7	0.0117
	V95%	91.0	98.9	0.0051
	V90%	96.2	99.7	0.0051
PW	V107%	0.9	10.7	0.0069
	V95%	93.7	99.0	0.0051
	V90%	96.7	99.8	0.0051

FB = free breathing; FIF = field-in-field; IN = light inhalation; PTVeval = evaluated planning target volume; PW = physical wedge; V107%, V95%, and V90% = percentage of PTVeval volume receiving ≥ 107%, ≥ 95%, and ≥ 90% of the prescription dose.
